# The Relative Weight of Temporal Envelope Cues in Different Frequency Regions for Mandarin Disyllabic Word Recognition

**DOI:** 10.3389/fnins.2021.670192

**Published:** 2021-07-15

**Authors:** Zhong Zheng, Keyi Li, Yang Guo, Xinrong Wang, Lili Xiao, Chengqi Liu, Shouhuan He, Gang Feng, Yanmei Feng

**Affiliations:** ^1^Department of Otolaryngology-Head and Neck Surgery, Shanghai Jiao Tong University Affiliated Sixth People’s Hospital, Shanghai, China; ^2^Shanghai Key Laboratory of Sleep Disordered Breathing, Shanghai, China; ^3^Sydney Institute of Language and Commerce, Shanghai University, Shanghai, China; ^4^Ear, Nose, and Throat Institute and Otorhinolaryngology Department, Eye and ENT Hospital of Fudan University, Shanghai, China; ^5^Department of Otolaryngology, Qingpu Branch of Zhongshan Hospital Affiliated to Fudan University, Shanghai, China; ^6^The First Affiliated Hospital of Jinzhou Medical University, Jinzhou, China

**Keywords:** relative weight, envelope cues, frequency region, Mandarin Chinese, disyllabic word

## Abstract

**Objectives:**

Acoustic temporal envelope (E) cues containing speech information are distributed across all frequency spectra. To provide a theoretical basis for the signal coding of hearing devices, we examined the relative weight of E cues in different frequency regions for Mandarin disyllabic word recognition in quiet.

**Design:**

E cues were extracted from 30 continuous frequency bands within the range of 80 to 7,562 Hz using Hilbert decomposition and assigned to five frequency regions from low to high. Disyllabic word recognition of 20 normal-hearing participants were obtained using the E cues available in two, three, or four frequency regions. The relative weights of the five frequency regions were calculated using least-squares approach.

**Results:**

Participants correctly identified 3.13–38.13%, 27.50–83.13%, or 75.00–93.13% of words when presented with two, three, or four frequency regions, respectively. Increasing the number of frequency region combinations improved recognition scores and decreased the magnitude of the differences in scores between combinations. This suggested a synergistic effect among E cues from different frequency regions. The mean weights of E cues of frequency regions 1–5 were 0.31, 0.19, 0.26, 0.22, and 0.02, respectively.

**Conclusion:**

For Mandarin disyllabic words, E cues of frequency regions 1 (80–502 Hz) and 3 (1,022–1,913 Hz) contributed more to word recognition than other regions, while frequency region 5 (3,856–7,562) contributed little.

## Introduction

The World Health Organization estimates that > 5% of the world’s population (approximately 466 million people) suffer from disabling hearing loss (WHO, 2020). Approximately one-third of people over the age of 65 years suffer from different degrees of sensorineural hearing loss (SNHL), one of the most common forms of hearing loss (WHO, 2020). Cochlear implants (CIs) remain the only effective device for restoring speech communication ability in patients with severe to profound SNHL (Tavakoli et al., 2015). In people with normal hearing, the cochlea converts speech signals into bioelectrical signals, which are transmitted through the auditory nerve to the brain so that listeners are able to sense various sounds. The basilar membrane in the cochlea can be regarded as a series of overlapping bandpass filters, each of which has its own unique characteristic frequency. When the basilar membrane is vibrated by sound, the sound signal of a characteristic frequency causes amplitude to peak at the corresponding basilar membrane partition (Smith et al., 2002). As electronic devices, CIs stimulate the auditory nerve *via* amplitude-modulated pulses that carry important temporal information of speech signals.

Speech is a complex acoustic signal and can be viewed in terms of two domains: temporal information and spectral information. Temporal information refers to information in speech signals with time-varying wave rates, which can be divided into the temporal envelope (E) below 50 Hz, periodic fluctuations in the range of 50–500 Hz, and temporal fine structure in the range of 500–10,000 Hz (Rosen, 1992). E cues contain temporal modulation information, which is most important for speech perception in quiet conditions, whereas the temporal fine structure can provide information in noisy environments and for tonal and pitch recognition (Smith et al., 2002; Xu and Pfingst, 2003; Moore, 2008; Ardoint and Lorenzi, 2010; Wang et al., 2016). Vocoder studies have shown that E modulation rates of 4–16 Hz are most important for speech intelligibility in quiet (Drullman et al., 1994a,b; Shannon et al., 1995). However, when speech has to be perceived against interfering speech, both slower and faster modulation rates, which are associated with prosodic (Füllgrabe et al., 2009) and fundamental frequency (Xu et al., 2002; Stone et al., 2008), respectively, become important for identification.

Recently, many researchers have investigated the relative importance of E cues from different frequency regions. Shannon et al. (2002) studied the influence of temporal E cues from different frequency regions on English recognition by removing specific spectral information. They found that the removal medium and high frequencies had greater impacts than low frequencies. This is supported by Apoux and Bacon (2004) who also reported that temporal E cues from different frequency regions are important in quiet conditions, while the high-frequency region (>2,500 Hz) is more important in noisy environments. In addition, using a classic high-pass and low-pass filtering experiment paradigm (French and Steinberg, 2005), Ardoint et al. (Ardoint and Lorenzi, 2010) demonstrated that E cues for frequency bands approximately 1,000–2,000 Hz are most important in French vowel–consonant–vowel speech recognition. The different frequency ranges and their respective importance in these investigations of non-tonal languages motivated us to examine the relative weight of E cues in different frequency regions for Mandarin Chinese speech recognition.

Mandarin Chinese is a tonal language and has the most first-language speakers of any language in the world. It includes 23 consonants, 38 vowels, and 5 tones. The 38 vowels consist of 9 monophthongs, 13 diphthongs and triphthongs, and 16 nasal finals. The tones include tone 1 (high-level), tone 2 (mid-rising), tone 3 (low-dipping), and tone 4 (high-falling) (Xu and Zhou, 2011). In addition, there is a fifth tone, usually called a neutral tone or tone 0, that occurs in unstressed syllables in multisyllabic words or connected speech (Yang et al., 2017). There are many polysyllabic words in Mandarin, most of which are disyllabic, and different tones can represent many different meanings (Nissen et al., 2005). Thus, the recognition of disyllabic words plays an important role in Mandarin speech recognition. Despite the large number of people who speak Mandarin as a mother tongue, there has been little emphasis on the relative weight of temporal and spectral information in different frequency regions for Mandarin Chinese. The present study intends to fill this knowledge gap. Our results may benefit CI wearers whose native language is Mandarin Chinese and ultimately improve their speech recognition performance and quality of life (McRackan et al., 2018).

Speech recognition is an interactive process between the speech characteristics of auditory signals and the long-term language knowledge of the listener, enabled by the decoding of speech through integrative bottom-up and top-down processes (Tuennerhoff and Noppeney, 2016). Speech recognition includes phonemes, syntax, and semantic recognition (Etchepareborda, 2003; Desroches et al., 2009). Phonemes are the smallest unit of sound that distinguish one word from another word in a language. Syntax refers to the meaning and interpretation of words, signs, and sentence structure, and depends on elements such as language environment and contextual information. Based on the semantic information of language, we can roughly judge the content range. Bottom-up mechanisms include phoneme recognition (Tuennerhoff and Noppeney, 2016), which disyllabic word recognition primarily relies on. Top-down mechanisms include syntactic and semantic information (Etchepareborda, 2003; Desroches et al., 2009). In an fMRI study, Tuennerhoff and Noppeney (2016) found that activations of unintelligible fine-structure speech were limited to the primary auditory cortices, but when top-down mechanisms made speech intelligible, the activation spread to posterior middle temporal regions, allowing for lexical access and speech recognition. Sentences can provide listeners with an envelope template where lexical and phonological constraints can help segment the acoustic signal into larger comprehensible temporal units, similar to the spatial “pop-out” phenomenon in visual object recognition (Dolan et al., 1997). Both top-down and bottom-up mechanisms are essential in the recognition of sentences.

While Mandarin Chinese word recognition relies more on tone recognition, sentence recognition can be inferred from context, which is consistent with the top-down mechanisms of speech recognition. Our team previously found that acoustic temporal E cues in frequency regions 80–502 Hz and 1,022–1,913 Hz contributed significantly to Mandarin sentence recognition (Guo et al., 2017). The present study builds on these findings and further investigates the relative weights of E cues for Mandarin disyllabic word recognition.

## Materials and Methods

### Participants

We recruited a total of 20 participants (10 males and 10 females), who were graduates of Shanghai Jiao Tong University with normal audiometric thresholds (≤ 20 dB HL) bilaterally at octave frequencies of 0.25–8 kHz. Their ages ranged from 22 to 28 (average, 24) years. All participants were native Mandarin speakers with no reported history of ear disease or hearing loss. Pure-tone audiometric thresholds were measured with a GSI-61 audiometer (Grason-Stadler, Madison, WI, United States) using standard audiometric procedures. No participant had any preceding exposure to the speech materials used in the present study. All participants provided signed consent forms before the experiment and were compensated on an hourly basis for their participation. The protocol was approved by the ethics committee of Shanghai Jiao Tong University Affiliated Sixth People’s Hospital (ChiCTR-ROC-17013460), and the experiment was performed in accordance with the Declaration of Helsinki.

### Signal Processing

Mandarin disyllabic speech test materials issued by the Beijing Institute of Otolaryngology were used for disyllable word recognition in quiet conditions. The test materials included 10 lists, each of which contained 50 disyllabic words covering 96.65% of the words used in daily life (Wang et al., 2007). The disyllable words were filtered into 30 contiguous frequency bands using zero-phase, third-order Butterworth filters (18 dB/octave slopes), ranging from 80 to 7,562 Hz (Li et al., 2016). Each band had an equivalent rectangular bandwidth for normal-hearing participants, simulating the frequency selectivity of the normal auditory system (Glasberg and Moore, 1990).

The E cues were extracted from each band using Hilbert decomposition followed by low-pass filtering at 64 Hz with a third-order Butterworth filter. E cues were then used to modulate the amplitude of a white-noise carrier. The modulated noise was filtered using the same bandpass filters and summed across frequency bands to form the frequency regions of acoustic E cues. We focused on the parameters used in clinics to assess hearing levels; i.e., low-frequency (< 500 Hz), medium-low-frequency (500–1,000 Hz), medium-frequency (1,000–2,000 Hz), medium-high-frequency (2,000–4,000 Hz), and high-frequency (4,000–8,000 Hz) regions. We chose cutoff frequencies closest to the audiometric frequencies 500, 1,000, 2,000, 4,000, and 8,000 Hz. Thus, frequency bands 1–8, 9–13, 14–18, 19–24, and 25–30 were combined to form frequency regions 1–5 (Table 1).

**TABLE 1:** Cutoff frequencies of the 30 frequency bands.

**Frequency regions**	**Band**	**Lower frequency (Hz)**	**Upper frequency (Hz)**
1	1	80	115
	2	115	154
	3	154	198
	4	198	246
	5	246	300
	6	300	360
	7	360	427
	8	427	502
2	9	502	585
	10	585	677
	11	677	780
	12	780	894
	13	894	1,022
3	14	1,022	1,164
	15	1,164	1,322
	16	1,322	1,499
	17	1,499	1,695
	18	1,695	1,913
4	19	1,913	2,157
	20	2,157	2,428
	21	2,428	2,729
	22	2,729	3,066
	23	3,066	3,440
	24	3,440	3,856
5	25	3,856	4,321
	26	4,321	4,837
	27	4,837	5,413
	28	5,413	6,054
	29	6,054	6,767
	30	6,767	7,562

To investigate the role of different frequency regions in Mandarin disyllabic word recognition, participants were presented with acoustic E cues from two frequency regions (10 conditions, namely, Region 1 + 2, Region 1 + 3, Region 1 + 4, Region 1 + 5, Region 2 + 3, Region 2 + 4, Region 2 + 5, Region 3 + 4, Region3 + 5, and Region 4 + 5), three frequency regions (10 conditions, namely, Region 1 + 2 + 3, Region 1 + 2 + 4, Region 1 + 2 + 5, Region 1 + 3 + 4, Region 1 + 3 + 5, Region 1 + 4 + 5, Region 2 + 3 + 4, Region 2 + 3 + 5, Region 2 + 4 + 5, and Region 3 + 4 + 5), and four frequency regions (5 conditions, namely, Region 1 + 2 + 3 + 4, Region 1 + 3 + 4 + 5, Region 1 + 2 + 4 + 5, Region 1 + 2 + 3 + 5, and Region 2 + 3 + 4 + 5). To prevent the possible use of transitional band information (Warren et al., 2004; Li et al., 2015), the frequency regions including disyllabic word E cues and complementary frequency regions (i.e., noise-masking), were combined to present a speech-to-noise ratio of 16 dB. For example, the “Region 1 + 2” condition indicates that the participant was presented with a stimulus consisting of E cues for disyllabic words in frequency regions 1 and 2 with noise in frequency regions 3, 4, and 5. Similarly, “Region 1 + 2 + 3 + 4” indicates that the stimulus comprised acoustic temporal E cues in frequency regions 1, 2, 3, and 4 with noise in frequency region 5.

### Testing Procedure

None of the participants had previously participated in perceptual experiments testing acoustic temporal E cues. The participants were tested individually in a double-walled, soundproof room. All stimuli were delivered *via* HD 205 II circumaural headphones (Sennheiser, Wedemark, Germany) at approximately 65 dB SPL (range, 60–75 dB SPL).

About 30 min of practice was provided before the formal test. The practice vocabulary comprised 50 disyllabic words (i.e., one list of the test material). Words were first presented under the “full region” condition and then experimental stimuli were presented randomly. Feedback was given during practice. To familiarize participants with the stimuli, they were allowed to listen to words repeatedly for any number of times before moving on to the next word until their performance stabilized.

In the formal test, participants were allowed to listen to words as many times as they wanted. Most participants listened to each word two or three times before moving on. All conditions and corresponding material lists were presented in random order for each participant to avoid any order effect. Participants were encouraged to repeat words as accurately as possible and to guess if necessary. No feedback was provided during the test. Each keyword in the disyllabic word lists was rated as correct or incorrect, and the results were recorded as the percentage of correct words under different conditions. The participants were allowed to take breaks whenever necessary. Each participant required approximately 1.5–2 h to complete the set of tests.

### Statistical Analysis

Statistical analyses were conducted using the Statistical Package for Social Sciences version 22.0 (IBM Co., Armonk, NY, United States). Because of our small sample size, we used the Kruskal–Wallis test to analyze results from different test conditions for disyllabic word recognition. Pairwise comparison using results of different test conditions for disyllabic word recognition was performed with *post hoc* analysis (the Bonferroni test). The relative weights of the five frequency regions were calculated using the least-squares approach (Kasturi et al., 2002). The Mann–Whitney *U* test was used to compare the relative weights of the five frequency regions for Mandarin disyllabic word and sentence recognition.

## Results

### Scores for Mandarin Disyllabic Word Recognition Across Conditions Using E Cues

**FIGURE 1:**
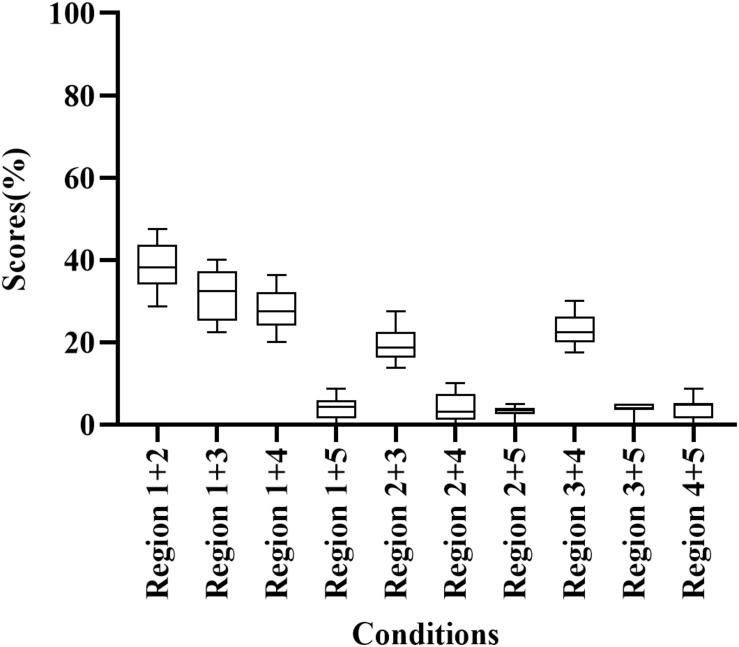
Percent-correct scores for disyllabic word recognition using acoustic temporal envelope in two-frequency-region conditions.

Participants correctly identified 3.13–38.13% of words when presented with E cues in two frequency regions (Figure 1); scores were highest for Region 1 + 2 (38.13% correct) and lowest for Region 2 + 4 (3.13% correct). Thus, these conditions were unfavorable for participants to understand the meaning of disyllabic words. In addition, the percentage of correct responses differed significantly among frequency region combinations (H = 167.288, *p* < 0.05). Regions 1 + 2 and Region 1 + 3 had significantly higher scores than other conditions with two frequency regions (adjusted *p* < 0.05).

**FIGURE 2:**
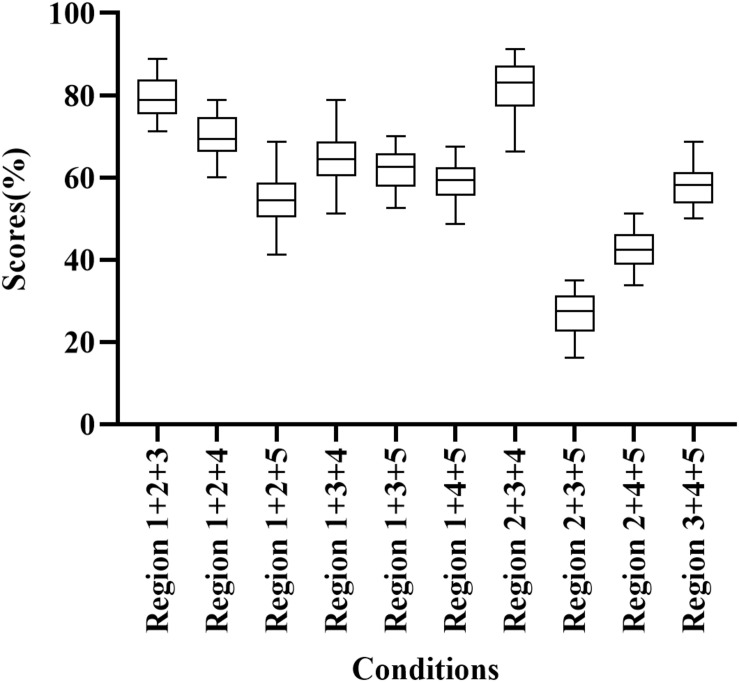
Percent-correct scores for disyllabic word recognition using acoustic temporal envelope in three-frequency-region conditions.

Participants correctly identified 27.50%–83.13% of words when presented E cues in three frequency regions (Figure 2). Region 1 + 2 + 3 and Regions 2 + 3 + 4 had high scores (78.75% and 83.13% correct, respectively), while Region 2 + 3 + 5 scored relatively low (27.50% correct). In addition, the percentage of correct responses differed significantly among frequency region combinations (H = 168.938, *p* < 0.05). Region 1 + 2 + 3 and Region 2 + 3 + 4 had significantly higher scores than other conditions with three frequency regions (adjusted *p* < 0.05).

**FIGURE 3:**
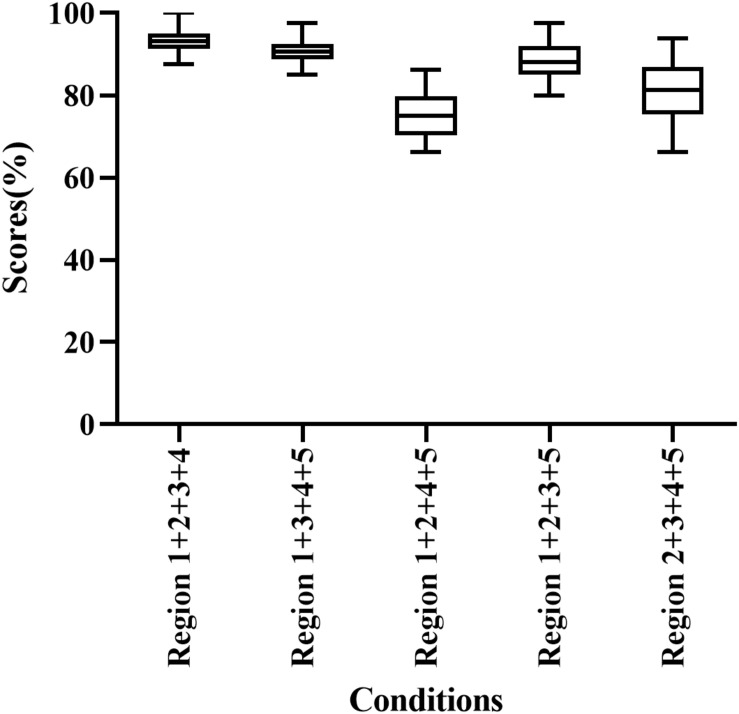
Percent-correct scores for disyllabic word recognition using acoustic temporal envelope in four-frequency-region conditions.

Participants correctly identified 75.00–93.13% of words when presented with E cues in four frequency regions (Figure 3). Region 1 + 2 + 3 + 4 had the highest score among all conditions (93.13% correct), while Region 1 + 2 + 4 + 5 had the lowest score among combinations of four frequency regions (75.00% correct). In addition, the percentage of correct responses differed significantly among frequency region combinations (H = 60.762, *p* < 0.05). As the number of frequency regions increased, speech recognition scores increased and the magnitude of the difference between the groups decreased. Region 1 + 2 + 3 + 4 and Region 1 + 3 + 4 + 5 had significantly higher scores than other conditions with four frequency regions (adjusted *p* < 0.05).

### Relative Weights of the Five Frequency Regions in Mandarin Disyllabic Word and Sentence Recognition

**FIGURE 4:**
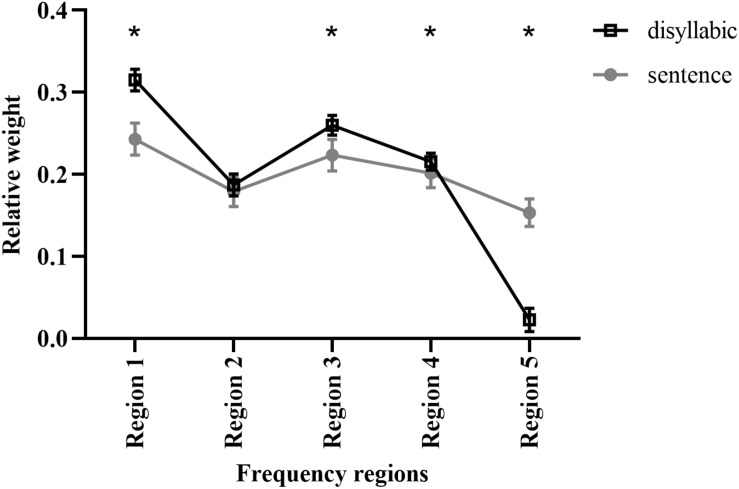
The relative weights of different frequency regions for Mandarin disyllabic word and sentence recognition using acoustic temporal envelope. The data for Mandarin sentence recognition was adopted from a previous study (Guo et al., 2017). The error bars represent standard errors. * Statistically significant (*p* < 0.05).

The relative weights of the five frequency regions for Mandarin disyllabic word recognition using acoustic temporal E cues were calculated using the least-squares approach (Kasturi et al., 2002). The strength of each frequency region was defined as a binary value (0 or 1) depending on whether the frequency region was present. The weight of each frequency region was then calculated by predicting the participant’s response as a linear combination of each frequency region’s intensity. The original weights for the five frequency regions of each participant were transformed to relative weights by summing their values, and each frequency region weight was represented as the original weight divided by the sum of all weights of the five frequency regions. Thus, the sum of the relative weights of the five frequency regions was equal to 1.0. The mean relative weights of frequency regions 1–5 were 0.31, 0.19, 0.26, 0.22, and 0.02, respectively (Figure 4). The relative weights differed significantly among frequency regions (H = 94.221, *p* < 0.05).

Our previous reports showed that the relative weights of the E cues from frequency regions 1–5 for Mandarin sentence recognition were 0.25, 0.18, 0.22, 0.20, and 0.15, respectively (Guo et al., 2017). Thus, the observed trends between sentence and disyllabic word recognition were similar. The E cues of frequency regions 1 and 3 contributed more to Mandarin disyllabic word and sentence recognition than those of the other frequency regions. Mandarin disyllabic word and sentence recognition had significantly different relative weights for frequency regions 1, 3, 4, and 5 (*p* < 0.05; Figure 4) but not for frequency region 2.

## Discussion

Vocoder technology is often used to simulate the signal processing of CIs when studying the function of E cues in speech recognition. The vocoder separates the speech signal into different frequency bands through bandpass filters. The E cues in different frequency bands are extracted and used to modulate white noise or sine waves. Eventually, the summed E cues of each frequency band are presented to the participants for perceptual experiments (Kim et al., 2015; Rosen and Hui, 2015). Previous research has shown that as the number of frequency bands segmented by a vocoder increases, the speech recognition rate of the subjects gradually increases (Shannon et al., 2004; Xu et al., 2005; Xu and Zheng, 2007). However, it is impossible to increase the number of intracochlear electrodes infinitely due to a number of constraints, including interference between adjacent electrodes (Shannon et al., 2004). Therefore, when the number of electrodes is fixed, it is necessary to study the relative importance of E cues in different frequency regions for speech recognition, especially given the trade-off between the number of spectral channels and the amount of temporal information (Xu and Pfingst, 2008).

It is worth noting that the bandpass filters used in the present study had a fairly shallow slope (18 dB/octave). The listening ability beyond the cutoff frequency that filtered the stimulus is called “off-frequency listening.” However, our normal-hearing participants may not have been able to efficiently use the speech cues in the off-frequency bands because, in vocoder processing, the fine structure of each frequency band is replaced by white noise.

The present study explored the relative importance of E cues across different frequency regions for Mandarin disyllabic word recognition. We found that E cues in different frequency regions contributed differentially to recognition scores. For Mandarin Chinese disyllabic words, frequency region 1 had the highest weight (0.31; Figure 4). Thus, the low-frequency region is most important for Mandarin recognition, which is consistent with a previous report on Mandarin Chinese sentence recognition (Guo et al., 2017). However, this is not consistent with the results of Ardoint et al., who found that E cues in higher regions (1,800–7,300 Hz) contributed more strongly to French consonant recognition (Ardoint et al., 2011). Explanations for this difference include differences in speech materials, since previous studies have shown that the type of speech material may have a strong impact on the use of acoustic temporal fine structure information (Lunner et al., 2012); the cutoff frequencies defined by different frequency regions used in different experiments; the methods of processing stimuli and evaluating weights; and, most importantly, differences inherent to the languages themselves. As a tone language, Mandarin Chinese has disyllabic words with different tones that can contain different lexical meanings (Fu et al., 1998; Wei et al., 2004). Fundamental frequency (F0) and its harmonics are the primary cues for lexical tone recognition, and tone contours play an important role in tonal language speech intelligibility (Feng et al., 2012). Kuo et al. (2008) found that when F0 information is provided, participants consistently have tone recognition rates of > 90%; however, without F0 information, E cue information contributes to tone recognition to a lesser extent. Considering that F0 information is mainly in the low-frequency region, which plays an important role in tone recognition (Wong et al., 2007), the low-frequency region (i.e., region 1) likely has a strong influence on the recognition of Mandarin Chinese.

The E cues information in the intermediate frequencies (i.e., region 3, 1,022–1,913 Hz) is important for speech recognition, which is consistent with previous results. Kasturi et al. (2002) found that when E cues in a band centered at 1,685 Hz were removed, English vowel and consonant recognition declined. In addition, Ardoint et al. (Ardoint and Lorenzi, 2010) found that E cues conveyed important distinct phonetic cues in frequency regions between 1,000 and 2,000 Hz. These results indicate that E cues in the frequency band 1,000–2,000 Hz are important for speech recognition regardless of language.

We found that relative weights differed significantly between Mandarin disyllabic word and sentence recognition in frequency regions 1, 3, 4, and 5 (*p* < 0.05) but not in frequency region 2. This may have been due to differences in speech materials as described earlier and the fact that tone plays a more important role in understanding disyllabic words (Feng et al., 2012; Wong and Strange, 2017). Auditory speech input is rapidly and automatically bound by the participant into a speech representation in a short-term memory buffer. If this information matches the speech representation in long-term memory, relatively automatic and effortless lexical acquisition occurs. Mismatches are effortlessly controlled using higher-level linguistic knowledge such as semantics or syntactic contexts. Therefore, when bottom-up processes fail, controlled processing and sentence contextual information are used (Rönnberg et al., 2013). It should be noted that relationship between working memory (cognitive processing) and speech-in-noise intelligibility is less evident for younger participants than older hearing-impaired participants (Füllgrabe and Rosen, 2016). In this study, a common feature of most sentence-recognition models is that long-term language knowledge helps the participant choose the appropriate phonological and lexical candidates, thereby allowing the participant to make the correct selection step by step based on the acoustic speech characteristics of the speech signal (McClelland and Elman, 1986; Norris et al., 2016). The results of this study showed that Mandarin disyllabic recognition relies more on tone recognition, which is consistent with a bottom-up mechanism, whereas sentence recognition is inferred from context, which is consistent with the top-down mechanism of speech recognition.

We found that Region 1 + 2 has the highest score among two-frequency regions. In addition, Region 2 + 3, Region 2 + 3 + 4, and Region 1 + 2 + 4 also yielded high scores; however, Region 2 had the second-lowest relative weight. Warren et al. (1995) reported that regions centered at 370 and 6,000 Hz strongly synergize but provide little information when presented separately. Healy et al. (Healy and Warren, 2003) also found that unintelligible individual speech regions became comprehensible when combined. We assessed participant performance under all possible combinations of frequency regions and found that frequency region 2 had synergistic effects when combined with adjacent regions (i.e., frequency regions 1 or 3), which led to increased word recognition scores.

This study had some limitations. First, participants were well educated, and the influence of education level has not been evaluated in previous studies. In addition, since cognitive abilities are associated with changes in speech processing with age (even in the absence of audiometric hearing loss) (Füllgrabe et al., 2014), our findings may not be directly applicable to CI performance in different age groups. Future studies are needed to evaluate the factors of age, education level, and cognitive ability.

Overall, E cues contained in low-frequency spectral regions are more important in quiet environments for Mandarin disyllabic word recognition than for non-tonal languages such as English. These differences may be determined by the tone characteristics of Mandarin Chinese, but the influence of signal extraction parameters, test materials, and test environments cannot be excluded.

## Conclusion

1. We found that E cues in frequency regions 1 (80–502 Hz) and 3 (1,022–1,913 Hz) significantly contributed to Mandarin disyllabic word recognition in quiet.

2. In contrast to English speech recognition, the low-frequency region contributed strongly to Mandarin Chinese disyllabic word recognition due to the tonal nature of the language.

## Data Availability Statement

The original contributions presented in the study are included in the article/supplementary material, further inquiries can be directed to the corresponding author/s.

## Ethics Statement

The studies involving human participants were reviewed and approved by the studies involving human participants were reviewed and approved by Ethics Committee of the Sixth People’s Hospital affiliated to the Shanghai Jiao Tong University. The patients/participants provided their written informed consent to participate in this study.

## Author Contributions

ZZ: conceptualization and writing—original draft. KL and YG: methodology and data curation. XW, LX, and CL: investigation. SH: supervision. GF: validation and project administration. YF: conceptualization, resources, writing—review and editing, and funding acquisition. All authors contributed to the article and approved the submitted version.

## Conflict of Interest

The authors declare that the research was conducted in the absence of any commercial or financial relationships that could be construed as a potential conflict of interest.
